# Prevalence and determinants of intention to use modern contraceptives among grand-multiparous women in sub-Saharan Africa

**DOI:** 10.1186/s13690-022-01006-x

**Published:** 2022-12-03

**Authors:** Oluwatobi Abel Alawode, Sylvester Reuben Okeke, Rajeeb Kumar Sah, Obasanjo Afolabi Bolarinwa

**Affiliations:** 1 Department of Sociology and Criminology & Law, University of Florida, Gainesville, FL 32611 USA; 2 Centre for Social Research in Health, UNSW Sydney, Sydney, Australia; 3 School of Human and Health Sciences, University of Huddersfield, Queensgate, Huddersfield, HD1 3DH UK; 4 Department of Public Health Medicine, School of Nursing and Public Health, University of KwaZulu-Natal, Durban, South Africa; 5 Department of Global Public Health, School of Allied and Public Health Professions, Canterbury Christ Church University, Canterbury, CT1 1QU UK

**Keywords:** Modern contraceptives use, Grand-multiparous women, Sub-Saharan Africa, Public health, DHS

## Abstract

**Background:**

Sub-Saharan Africa, characterised by high fertility and low contraceptive use prevalence, remains one of the settings with the poorest maternal and child health indices globally. Studies have established that grand-multiparous women are at increased risk of these adverse maternal health outcomes, and contraceptive use is important to averting these adverse outcomes. Thus, this study examines the prevalence and determinants of intention to use modern contraceptives among grand-multiparous women in 10 sub-Saharan African countries with high fertility rates.

**Materials and methods:**

The study utilized data from the last installments of the Demographic and Health Survey from the 10 leading countries with the highest total fertility rates in sub-Saharan Africa. These countries include: Angola, Benin, Burundi, Chad, Cote d’Ivoire, the Republic of the Congo, Democratic Republic of Congo, Mali, Niger, and Nigeria. Data analysis of 23,500 grand-multiparous women was done at three univariate levels involving a frequency table and bar chart. We employed bivariate logit and multivariate logit regression at the bivariate and multivariate levels to achieve the study objectives. A significant level was determined at *p* < 0.05.

**Results:**

Our study found that less than 40% of grand-multiparous women in these high fertility countries in sub-Saharan Africa, have the intention to use modern contraceptives (39%), but country variations exist with as low as 32.8% in Angola to as high as 71.2% in the Republic of the Congo. The study found that modern contraceptives use intention among grand-multiparous women in these high fertility countries was predicted by a history of contraceptive use and pregnancy termination, exposure to family planning messages on social media, and knowledge of family planning methods. Others were women’s fertility planning status, ideal family size, number of marriages (remarriage), couple’s fertility desire, current age, and level of education.

**Conclusion:**

In the high fertility context of sub-Saharan Africa, characterized by low contraceptive use, improving contraceptive use intention among grand-multiparous women is vital for preventing adverse maternal and child health outcomes, including mortality, resulting from a high-risk pregnancy. Hence, interventions should be more innovative in targeting this group of women to increase the contraceptive prevalence rate in line with Family Planning 2030 goals, and ultimately reduce high fertility rates in the region.

## Background

Sub-Saharan Africa (SSA) remains one of the settings with the poorest maternal and child health indices globally [1–3]. Available data indicates that though progress is being made globally in reducing these indicators, the trend of the current progress is still less satisfactory. United Nations (UN) inter-agency estimate indicates that the global maternal mortality ratio declined by 38% between 2000 and 2017, from 342 deaths to 211 deaths per 100,000 live births. However, this decline representing a 2.9% annual drop, falls short of the 6.4% annual decline rate required to achieve the Sustainable Development Goal (SDG) 3.1 of 70 maternal deaths per 100,000 live births by 2030 [3, 4]. Less than a decade to 2030, the global maternal mortality ratio is still high, and SSA accounts for the largest proportion of this ratio, thereby necessitating greater research-oriented efforts in this setting if the SDG targets are to be achieved. This is more so considering the significant threat that the disruptions associated with the current 2019 coronavirus disease pandemic may have on maternal and child health services and programmes [5–7]. In the face of this urgent task, evidence-based policies, and programmes to accelerate strategies such as the utilization of modern contraceptives, which are effective in improving maternal and child health [8–10], are crucial.

Though studies have explored contraceptives use and associated factors among women of reproductive health age in SSA [11–13], we look more closely at grand-multiparous women, a sub-population at greater risk for adverse maternal and child health outcomes [14, 15]. Despite the vulnerability of this sub-population to pregnancy-related complications, evidence about their contraceptive use intention and determinants are still emerging [12]. Grand multiparity, which has been linked to numerous adverse maternal health outcomes [15, 16], is common among women in SSA [11, 15, 17], where a high fertility rate coexists with low contraceptives use [18, 19]. Therefore, we focus on 10 sub-Saharan African countries with the highest level of fertility [20]. This way, we may better understand how tailored services and programmes can be designed and implemented to reach this most vulnerable sub-population.

A large body of evidence has consistently identified the importance of contraceptives in preventing adverse reproductive health outcomes [11, 14]. Recognising controversies surrounding the definition of grand multiparity in literature [14, 15], we adopted the measure used in related studies in which grand multiparity is defined as women with at least five live births [16, 21, 22]. Though grand multiparity has been described as low and high in developed and developing countries, respectively [15], a recent retrospective cohort study of three decades of data (1989–2018) indicates that grand multiparty is increasing in the United States and that this increase is positively correlated with adverse pregnancy outcomes [16]. Moreover, both maternal and neonatal-perinatal complications have also been associated with grand multiparity in retrospective cross-sectional, retrospective case-control and comparative cross-sectional studies in SSA countries [15–17]. Recent evidence from the United States shows that grand multiparity induces hypertensive disorders during pregnancy [16]. There are potential concerns if a similar trend exists in SSA, where maternal health services and contraceptives are less utilised, mothers will be at higher risk of death. Understanding how to improve modern contraceptive use among grand-multiparous women is important in addressing the adverse indicators of maternal and child health in SSA and achieving SDG 3.

Based on the Theory of Planned Behaviour, intention is an important factor in health behaviour [23–25]. Though intention may not necessarily guarantee behaviour, it is unlikely that health behaviour change will occur without intention [23, 26]. As barriers that might be impeding the utilisation of modern contraceptives––such as availability, acceptability, and accessibility––are being identified and addressed [27, 28], understanding behavioural intention to use these contraceptives––and among a highly vulnerable sub-population––is equally important. Evidence leading to the development of the Health Belief Model indicates that addressing barriers to positive health behaviour does not guarantee undertaking such behaviour [29]. Consequently, understanding the intention to use modern contraceptives and its determinants might be an important step towards improving contraceptive use among all reproductive-aged women, especially multiparous women.

## Methods and materials

### Data source and study design

This study utilized data from the last installment of the Demographic and Health Surveys (DHS) for 10 SSA countries with the highest fertility rates. The DHS is a nationally representative survey conducted by the statistical or population agency in more than 95 countries across the world with technical assistance from ICF Macro International and funding provided by the United States Agency for International Development (USAID) to elicit information on the demographic and health characteristics of the population in these countries with the view to providing evidence for program planning and implementation. These surveys were all conducted between the years 2012 and 2018. These surveys’ methods and data collection procedures have been published elsewhere [30]. The DHS data were designed to be nationally representative, cross-sectional, household sample surveys with large sample sizes, typically between 5000 and 45,000 [13, 31].

### Data collection and sampling procedures

The sample design for the survey involves a multistage design that selected and interviewed separately nationally representative probability sample of women aged 15–49 years, using strata for rural and urban areas and different regions of individual countries. The survey questionnaires are similar across countries and were administered by interviewers to participants across all survey countries, yielding comparable data. Only countries with TFR greater than 5.1 were included in this study; these countries also have a high percentage of women who have had 5 or more children (grand-multiparous). The included 10 countries in this study were: Angola, Benin, Burundi, Chad, Cote D’Ivoire, the Republic of the Congo (Congo), Democratic Republic of Congo (Congo DR), Mali, Niger, and Nigeria [22] as indicated in Table 1. This inclusion criterion resulted in a total sample size of 23,500 women, which is in line with the previous studies [15, 22].

**Table 1:** Distribution of the study sample by countries

Countries	Survey Year	Weighted (n)	Weighted (%)	TFR
Angola	2015–16	1686	7.17	6.2
Benin	2018–19	1969	8.38	5.7
Burundi	2016–17	2148	9.14	5.5
Cote D’Ivoire	2011–12	826	3.51	4.8
Chad	2014–15	3448	14.67	5.6
Congo	2011–12	496	2.11	5.1
Congo DR	2013–14	2056	8.75	6.6
Mali	2018	1799	7.66	6.3
Niger	2012	2848	12.12	7.6
Nigeria	2018	6224	26.49	5.3
All countries		23,500	100.00	5.9

*TFR* Total Fertility Rate, *Source* DHS

### Definition of study variable

#### Outcome variable

The outcome variable in the study is intention to use modern contraceptives among grand-multiparous in SSA. The response to the question on intention to use modern contraceptive was asked during the DHSs and the responses were categorised as “use later”, “unsure about use” and “does not intend to use”. We focused on ‘use later’ and ‘does not intend to use as it allows for more focus and direction for practical implications and interventions, in consistency with previous studies [32, 33]. Hence, those grand-multiparous women who reported “does not intend to use later” were coded as 0, and those “who stated intention to use later” were coded 1. Consequently, grand-multiparous women who reported that they were “unsure about the use of modern contraceptives” were excluded from this study analysis.

### Explanatory variables

The explanatory variables were selected based on the theory of planned behaviour and what has been done in previous studies [22, 25, 34]. Place of residence was recoded as urban (1) and rural (2), age of the woman as 20–24 (1), 25–34 (2), 35–49 (3), Age at first birth- 8-19 (1), 20–29 (2), 30+ (3). We also included women’s type of marriage, which was coded as monogamous (1) and polygamous (2), this is followed by a variable on the ideal number of children that was a count variable and a non-numeric response. Women with the ideal number of children that were 5 or less were coded as “1” Whilst those with 5 or more and those with non-numeric responses were coded as “2”, this measurement is based on previous studies where it has been suggested that women with non-numeric responses invariably prefer large family sizes [35]. Fertility desire of couple was recoded as 1 – Both want same, 2 – Husband wants more than woman, 3 – Husband wants fewer than woman and 4 - Don’t know. Woman’s level of education were coded as No education (0), primary (1), secondary (2), and higher (3). History of contraceptive use, which refers to whether the woman has used contraceptive in the past or not was coded as ever used (1) and never used (2). History of pregnancy termination was coded as No (1) and Yes (2). The planning status of their last pregnancy was coded wanted (1), mistimed (2), and unwanted (3). For exposure to family planning messages, it was coded as No exposure (1) and exposed (2), Household power relation was developed from a list of variables that measured women empowerment and household decision making, and we arrived at whether a woman is involved (1) and woman not involved (2). These variables include the involvement of women in healthcare decisions making, important household purchases, the decision to visit relatives, and the decision on household earnings. For these decision-making variables, a woman was regarded as involved if she reported that she makes decisions alone or alongside her partner. Child mortality experience was coded as No experience (2) and experienced (2). In addition, we considered the number of marriages a grand-multiparous woman has ever had and coded the responses as once (1) and more than once (2), work status of the women was coded as No (1) and Yes (2) while knowledge of family planning method was coded as No (1) and Yes (2).

### Statistical analysis

Data management and analysis were implemented using Stata version 16 software. Descriptive statistics were used to summarize demographic characteristics, economic characteristics, marriage type, household structures, parity, fertility desire and preferences, and child death experience. Dataset was weighted by applying the recommended weight command to avoid over-sampling and non-response adjustment. At the bivariate level, we conducted a bivariate logistic regression to assess the association between each variable and contraceptive use intention among grand-multiparous women with their odds ratios (ORs) and their corresponding 95% confidence intervals (CIs) presented, and afterwards, a multivariable logistic regression test was performed to determine the adjusted likelihood of the determinants of the intention to use modern contraceptives. The multivariable logistics regression results were presented in adjusted odds ratio (aOR) with their corresponding 95% confidence intervals (CIs), and aOR less than 0.05 is considered statistically significant. The multicollinearity test, which used the variance inflation factor (VIF), revealed no collinearity among the explanatory variables used in this study.

### Ethical consideration

Our study is based on secondary data analysis with all the identifier information removed. The ethics committee approved the survey of the ORC Macro and the National Research Ethics Committee in individual countries. Also, study participants gave informed consent before they participated in the survey, and all information was collected with the promise of confidentiality.

## Results

**Fig. 1:**
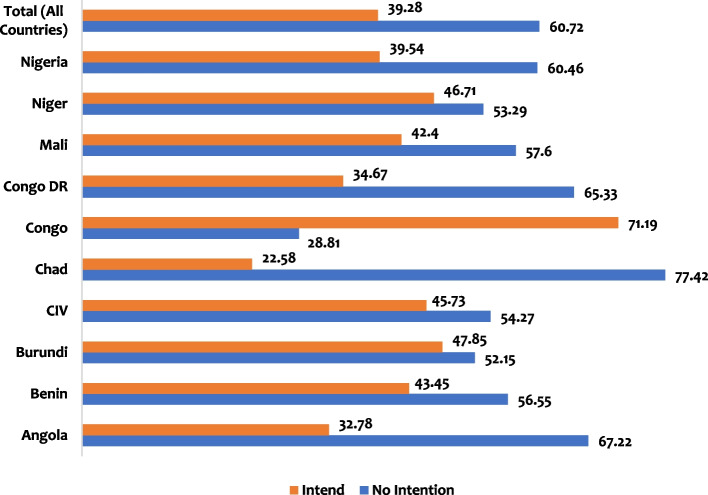
Proportion of Intention and Non-Intention to use Modern Contraceptives among Grand-multiparous women in 10 high fertility countries in sub-Saharan Africa

Intention to use modern contraceptives among grand-multiparous women is highest in Congo at 71%, while it is lowest in Chad, standing at approximately 23%. Generally, the intention to use modern contraceptives among this group of women is 39%, while about 61% do not intend to use modern contraceptives as indicated in Fig. 1.

It was found that more than half of the grand-multiparous women are between the age of 35 to 49 years (53%), while 46% are between 25 and 34 years and 1% are 20–24 years of age. About 78% are rural residents. Majority of the grand-multiparous women had their first child at less than 19 years of age (78%), 29% had their first birth between 20 and 29, while less than 1% had their first birth at after 30 years. 61% are living in monogamous marriages. For fertility preference, it can be reported that 77% of the women consider five or more children as the ideal number of children, and 23% stated that less than five children is the ideal number of children. In terms of fertility desire, 43% of the grand-multiparous women stated that their husband wants more children than the women, 23% of these grand-multiparous women reported that they want the same number of children as their husbands, while 6% stated that they want fewer children compared to their husbands.

The majority of these women have no formal education (68%), while less than 1% of the grand-multiparous women have higher education (0.8%). History of contraceptive use showed that 83% of the grand-multiparous women have never used a contraceptive method, while 17% reported ever using a method to avoid getting pregnant. Also, 83% have never terminated a pregnancy. It can also be reported that 81% wanted their last pregnancy, 8% did not want it, and 12% felt it was mistimed.

In addition, exposure to family planning messages is low among these groups of women, with 26% reporting that they have been exposed to mass media-based family planning messages in the past year while 74% reported non-exposure. 55% of these grand-multiparous women reported that they are involved in household decision-making, even as 45% reported that their partner makes all the decisions. It can also be reported that 58% of these women have experienced the death of a child. 84% have never remarried, while 16% have been in more than one marriage. The study also found that 66% of these grand-multiparous women work while 34% are not. The majority of the grand-multiparous women reported knowing a family planning method (76%), while 24% reported that they do not know any method of family planning.

Intention to use modern contraceptives is high among grand-multiparous women who are between the age of 25–34 (44%), those residing in urban areas (43%), and those who had their first birth at a very early age (40%), grand-multiparous women within monogamous marriages (60%), those with less than 5 ideal number of children (51%), women whose husbands want fewer children (48%), those with higher education (55%), those with history of pregnancy termination (45%), those whose last pregnancy was mistimed (57%), those with exposure to family planning messages (52%), women who are not involved in household decision making (43%), those who have experienced the death of a child (40%), those who have married more than once (46%), those currently working (43%), and those with knowledge of family planning (46%). Results from the bivariate association showed a statistically significant association between age, type of place of residence, ideal number of children, fertility desire, level of education, history of contraceptive use, history of pregnancy termination, fertility planning status, exposure to family planning messages, household power relations, number of marriages, work status, and knowledge of family planning method of these grand-multiparous women and intention to use modern contraceptive (Table 2).

**Table 2:** Intention to use modern contraceptives by respondent’s characteristic and bivariate logit regression of the determinants of modern contraceptives use intention among grand-multiparous women in sub-Saharan Africa

	Frequency (***n*** = 23,500)	Percentage (%)	Intention to Use Modern Contraceptive % [95% CI]	Bivariate logistic regression cOR [95% CI]
**Age**				
20–24 ^ref^	261	1.11	41.34 [35.74–47.18]	
25–34	10,756	45.77	43.51 [42.58–44.46]	1.09 [0.82–1.43]
35–49	12,483	53.12	35.65 [34.83–36.49]	0.73 *** [0.56–0.97]
**Type of place of residence**				
Urban ^ref^	5267	22.41	42.77 [41.45–44.10]	
Rural	18,233	77.59	38.26 [37.56–38.96]	0.76 *** [0.69–0.83]
**Age at first birth**				
8–19 ^ref^	16,472	70.09	39.55 [38.80–40.30]	
20–29	6893	29.33	38.87 [37.75–40.00]	0.96 [0.90–1.03]
30+	135	0.58	29.01 [22.53]	0.61 [0.42–0.91]
**Type of Marriage**				
Monogamous ^ref^	14,375	61.17	60.25 [59.45–61.04]	
Polygamous	9125	38.83	61.51 [60.50–62.51]	0.98 [0.91–1.05]
**Ideal Number of Children**				
5 or less ^ref^	5413	23.03	50.97 [49.62–52.31]	
5 or more	18,087	76.97	35.88 [35.19–36.58]	0.56 *** [0.52–0.61]
**Fertility Desire**				
Both want same ^ref^	5420	23.07	40.90 [39.62–42.19]	
Husband wants more	10,054	42.79	39.39 [38.43–40.34]	0.95 [0.87–1.04]
Husband wants fewer	1346	5.72	47.71 [44.99–50.44]	1.25 *** [1.07–1.47]
Don’t know	6680	28.43	36.18 [35.05–37.33]	0.81 *** [0.73–0.89]
**Maternal Level of Education**				
No Education ^ref^	16,069	68.38	34.69 [33.96–35.43]	
Primary	5284	22.49	46.39 [45.06–47.72]	1.54 *** [1.41–1.67]
Secondary	1969	8.38	54.28 [52.18–56.38]	1.97 *** [1.74–2.24]
Higher	178	0.76	54.55 [46.88–62.00]	1.78 *** [1.19–2.65]
**History of Contraceptive Use**				
Ever used ^ref^	4089	17.4	70.40 [68.98–71.77]	
Never used	19,411	82.6	32.78 [32.13–33.44]	0.23 *** [0.21–0.25]
**History of Pregnancy Termination**				
No ^ref^	19,547	83.18	38.00 [37.33–38.68]	
Yes	3953	16.82	45.45 [43.91–46.99]	1.23 *** [1.13–1.33]
**Fertility Planning Status**				
Wanted ^ref^	18,920	80.51	35.73 [35.05–36.41]	
Mistimed	2757	11.73	56.95 [55.08–58.79]	2.27 *** [2.06–2.50]
Unwanted	1823	7.76	49.57 [47.30–51.84]	1.66 *** [1.48–1.87]
**Exposure to Family Planning Messages**				
No Exposure ^ref^	17,444	74.23	35.38 [34.69–36.08]	
Exposed	6056	25.77	51.68 [50.37–52.98]	1.82 *** [1.67–1.98]
**Household Power Relations**				
Woman Involved ^ref^	12,986	55.26	36.25 [35.42–37.08]	
Woman Not Involved	10,514	44.74	42.93 [41.99–43.87]	1.28 *** [1.19–1.37]
**Experience of Child Death**				
No experience ^ref^	9858	41.95	38.44 [37.51–39.38]	
Experienced	13,642	58.05	39.91 [39.09–40.74]	1.04 [0.94–1.08]
**Remarriage**				
Once ^ref^	19,841	84.43	38.04 [37.37–38.71]	
More than once	3659	15.57	46.03 [44.42–47.64]	1.34 *** [1.22–1.47]
**Work Status**				
No ^ref^	7981	33.96	32.81 [31.79–33.85]	
Yes	15,519	66.04	42.56 [41.79–43.33]	1.35 *** [1.25–1.46]
**Knowledge of Family Planning Method**				
No ^ref^	5563	23.67	21.03 [20.05–22.06]	
Yes	17,937	76.33	45.88 [45.14–46.62]	3.05 *** [2.76–3.37]

*** *p* < 0.05, *cOR* Crude Odds Ratio, *CI* Confidence Interval, *ref* Reference category

Table 3 presents the multivariate logit regression analysis of the factors associated with the intention to use modern contraceptives among grand-multiparous women in the highest fertility countries in SSA. We present the full model, and the result showed that grand-multiparous women who have never used contraceptives have significantly lower odds of intending to use modern contraceptives [aOR = 0.30, CI = 0.27–0.33]. A statistically significant relationship was also found between fertility planning status and intention to use modern contraceptives among these grand-multiparous women, those whose last pregnancy was mistimed [aOR = 1.85, CI = 1.67–2.07] and unwanted compared to those who wanted their pregnancy [aOR = 1.47, CI = 1.27–1.68] have higher odds of intending to use modern contraceptive. Grand-multiparous women with a history of pregnancy termination have a higher likelihood of using modern contraceptives [aOR = 1.10, CI = 1.01–1.21]. The analysis also found that the odds of modern contraceptive use intention is high among grand-multiparous women who have been exposed to mass media-based family planning messages compared to those who are not exposed [aOR = 1.37, CI = 1.25–1.50], those with knowledge of a family planning methods have a higher likelihood of intending to use modern contraceptive compared to those with no knowledge [aOR = 2.31, CI = 2.09–2.56].

**Table 3:** Multivariate logistic regression results of the Intention to use modern contraceptives determinants among grand-multiparous women in sub-Saharan Africa

Variables	aOR [95% CI]	aOR [95% CI]	aOR [95% CI]
**History of Contraceptive Use**			
Ever used ^**ref**^			
Never used	0.29 *** [0.26–0.32]	0.30 *** [0.28–0.34]	0.30 *** [0.27–0.33]
**History of Pregnancy Termination**			
No ^**ref**^			
Yes	1.08 [0.99–1.18]	1.05 [0.96–1.15]	1.10 *** [1.01–1.21]
**Fertility Planning Status**			
Wanted ^**ref**^			
Mistimed	2.14 *** [1.93–2.38]	1.97 *** [1.77–2.19]	1.85 *** [1.67–2.07]
Unwanted	1.33 *** [1.26–1.65]	1.29 *** [1.12–1.48]	1.46 *** [1.27–1.68]
**Exposure to Mass Media Family Planning Messages**			
Not Exposed ^**ref**^			
Exposed	1.34 *** [1.23–1.47]	1.34 *** [1.23–1.47]	1.37 *** [1.25–1.50]
**Knowledge of Family Planning Method**			
No ^**ref**^			
Yes	2.33 *** [2.10–2.57]	2.27 *** [2.06–2.51]	2.31 *** [2.09–2.56]
**Ideal Family Size**			
Less than 5 ^**ref**^			
5 or more		0.80 *** [0.73–0.88]	0.80 *** [0.73–0.87]
**Remarriage**			
No ^**ref**^			
Yes		1.28 *** [1.16–1.41]	1.32 *** [1.20–1.46]
**Fertility Desire**			
Both want the same ^**ref**^			
Husband wants more		1.13 *** [1.03–1.24]	1.12 *** [1.01–1.23]
Husband wants fewer		1.14 [0.96–1.36]	1.13 [0.94–1.34]
Don’t know		1.04 [0.93–1.15]	1.02 [0.92–1.13]
**Household Power Relations**			
Woman involved ^**ref**^			
Woman not involved		1.04 [0.96–1.13]	1.08 [0.99–1.17]
**Highest Level of Education**			
No Education ^**ref**^			
Primary		1.34 *** [1.23–1.48]	1.34 *** [1.23–1.47]
Secondary		1.43 *** [1.25–1.63]	1.43 *** [1.25–1.64]
Higher		1.05 [0.69–1.58]	1.15 [0.77–1.71]
**Type of Place of Residence**			
Urban ^**ref**^			
Rural			1.04 [0.94–1.15]
**Age of Woman (Years)**			
20–24 ^**ref**^			
25–34			0.90 [0.68–1.19]
35–49			0.54 *** [0.41–0.72]
**Age at first birth (Years)**			
8–19 ^**ref**^			
20–29			0.99 [0.92–1.08]
30+			0.87 [0.57–1.35]

*** *p* < 0.05, *aOR* Adjusted Odds Ratio, *CI* Confidence Interval, *ref* Reference category

Furthermore, the likelihood of intending to use modern contraceptives among grand-multiparous women is significantly lower among those with five or more ideal numbers of children compared to those with less than five ideal numbers of children [aOR = 0.80, CI = 0.73–0.87], significantly higher among those with the same fertility desire as their husbands compared to those whose husband want fewer or more [aOR = 1.12, CI = 1.01–1.23], grand-multiparous women with primary [aOR = 1.34, CI = 1.23–1.47], secondary [aOR = 1.43, CI = 1.25–1.64] and higher education [aOR = 1.15, CI = 0.77–1.71] have a higher likelihood of intending to use modern contraceptives comparing to those with no education. It was also found that older grand-multiparous women that are 25–34 [aOR = 0.90, CI = 0.68–1.19] and 35–49 years old [aOR = 0.54, CI = 0.41–0.72] are significantly less likely to have the intention of using modern contraceptive compared to younger one between the age group of 20–24.

## Discussion

Considering that intention is a significant predictor of health behaviour [23] and that evidence around contraceptive use intention among grand-multiparous women in SSA is less understood, we utilized a set of reliable, valid, and representative multi-country data to explore this important population health issue. We found that contraceptive use intention is generally relatively low at 39%. Interestingly, over 3 in 5 of these grand-multiparous women do not have a formal education, about 4 in 5 have never used any form of contraceptive, while 3 in 4 did not receive any form of family planning message in the past year.

We found a low intention to use modern contraceptives (39%) among grand-multiparous women in SSA. This low level––though higher than the 19% reported among this sub-population in Nigeria [22] –– may indicate that existing policies and programmes to improve modern contraceptives use are either not effective for this sub-population or not reaching them at all. As established by the Theory of Planned Behaviour, though the intention to use modern contraceptives in the future may not guarantee actual use, future use may not occur if there is no intention to do so. Therefore, it is a concern that about 3 in 5 grand-multiparous women in SSA, who are highly vulnerable to adverse pregnancy outcomes, do not intend to use modern contraceptives. This high proportion of non-intending women, despite vulnerability, may therefore continue to reinforce a vicious cycle of adverse maternal and child health care as well as put avoidable pressure on existing and overstretched maternal and child health services [36, 37].

Our findings also showed groups within grand-multiparous women that may have a higher level of vulnerability to adverse pregnancy outcomes because of not intending to use modern contraceptives and require to be reached with tailored interventions. Grand-multiparous women who live in rural areas are less educated and not working fall under this category. Also, grand-multiparous women who are older (25 years and above), not receiving family planning messages, those in polygamous marriages, and those with a low level of family planning knowledge equally need such targeted interventions as they may also be less likely to use modern contraceptives. The lesser likelihood of rural grand-multiparous women reporting modern contraceptive use may be attributed to several intersecting reasons. This group of grand-multiparous are also more likely to be less educated, experience higher levels of patriarchal arrangement, and operate in a setting where fads and misconceptions about modern family planning could be higher. Targeting these women with interventions designed in line with these contextual issues could help improve their intention and actual use of modern contraceptives. For instance, family planning messages may not be restricted to ante-natal clinics as there may still be a below level of utilisation of these services, especially in rural areas [38–40].

A broader community-based approach involving community leaders and men and women groups in planning, implementation, and evaluation, may yield better results. Considering that the patriarchal system is common in SSA, restricting family planning messages to grand-multiparous women could be less effective. This is because imbibing messages from such an arrangement could distort the patriarchal social order where the man largely makes the decision. The broader community approach could provide an avenue for husbands of grand-multiparous women to gain first-hand information and clarify their misconceptions. Importantly, girl-child education must continuously be given adequate attention as people are better equipped to source, interpret, and utilise information better if they are educated. Previous evidence has linked education to the use of modern contraceptives [22] and their actual use among reproductive-age women [12, 18, 19, 32]. Educated women are also more likely to be in the workforce. Evidence abounds that working women have greater intention to use modern contraceptives [22], just as they are also less likely to become grand-multiparous [15]. Also, women who are educated up to secondary school or above are more likely to utilise antenatal care services [38] and this could further enhance maternal and child health care. Educating the girl child is thus an important strategy that could help in attaining maternal and child health goals and targets.

### Strengths and limitations

A key limitation of the study is the inability to establish causality due to the cross-sectional nature of the survey design, whose data was used in this study. It is also important to note that the study is based on self-reported information, which is prone to recall bias and social desirability bias, especially for information about the history of contraceptive use and exposure-related questions in the survey. However, our study is not devoid of strength. One strength of the study lies in the fact that the study utilized the latest nationally representative datasets from SSA countries characterized by high fertility and low contraceptive prevalence, which makes the study’s findings generalizable to this group of women who have been largely ignored in public health literature.

### Implications for policy and public health practice

We hereby offer some important implications for policy and practice based on the findings from this study. It is already established that family planning can avert the adverse maternal and child outcomes associated with pregnancy among these women; hence, family planning programs focused on these high fertility countries should target this subpopulation of women with innovative sensitization on the dangers of unplanned pregnancies for their health which could spur the intention and eventual usage of modern contraceptive among these group of women. Behaviour change communication targeting norms and efficacy around modern contraceptive use for this group of women is also important, and this should be the target of intervention programs in the study settings. In addition, our study found that exposure to mass media family planning messages increased the likelihood of intending to utilize modern contraceptives among this group of women. Although, this has been found in other studies and suggestions made for public health practice in this regard. However, it is important to state that these interventions should be more innovative in their health communication strategies aimed at disseminating family planning messages that will educate these women on the importance of modern contraceptives for their health. For policy, the findings call for policymakers’ urgent attention to policy frameworks that will strengthen and encourage interventions, which will help reduce unwanted and unintended pregnancies and clandestine abortions – a significant portion of these types of women have a history of pregnancy termination. Policy efforts should also be geared towards helping advocacy efforts of all concerned family planning stakeholders in these countries to achieve the Family Planning 2030 goals.

## Conclusion and recommendations

In a high fertility context such as SSA, where fertility rates are high, and the desire for more children is high, improving contraceptive use intention, and actual use of contraception among grand-multiparous women is beneficial for several reasons. This improvement could help prevent maternal morbidity and mortality that could result from a high-risk pregnancy, increase contraceptive prevalence rate in line with Family Planning 2030 goals, and ultimately reduce high fertility rates. Designing, implementing, and evaluating interventions informed by the contextual issues associated with non-intention to use modern family planning––such as poor exposure to family planning messages, low girl-child education, and residing in rural areas, among others––using a broader community-based approach is needed.

## Data Availability

The datasets utilized in this study can be accessed freely at https://dhsprogram.com/data/available-datasets.cfm.

## Abbreviations

SSA: Sub-Saharan Africa
DHS: Demographic and Health Survey
CI: Confidence Interval
AOR: Adjusted Odds Ratio
